# Supplementary material for: Comparison of different techniques for prehospital cervical spine immobilization: Biomechanical measurements with a wireless motion capture system
Source: PLoS One. 2023 Nov 28;18(11):e0292300. doi: 10.1371/journal.pone.0292300 (PMC10683997; doi:10.1371/journal.pone.0292300)
Supplement: S1 Appendix — (PDF) [file pone.0292300.s001.pdf]

Technique P1S0: rescue sheet without rigid cervical collar

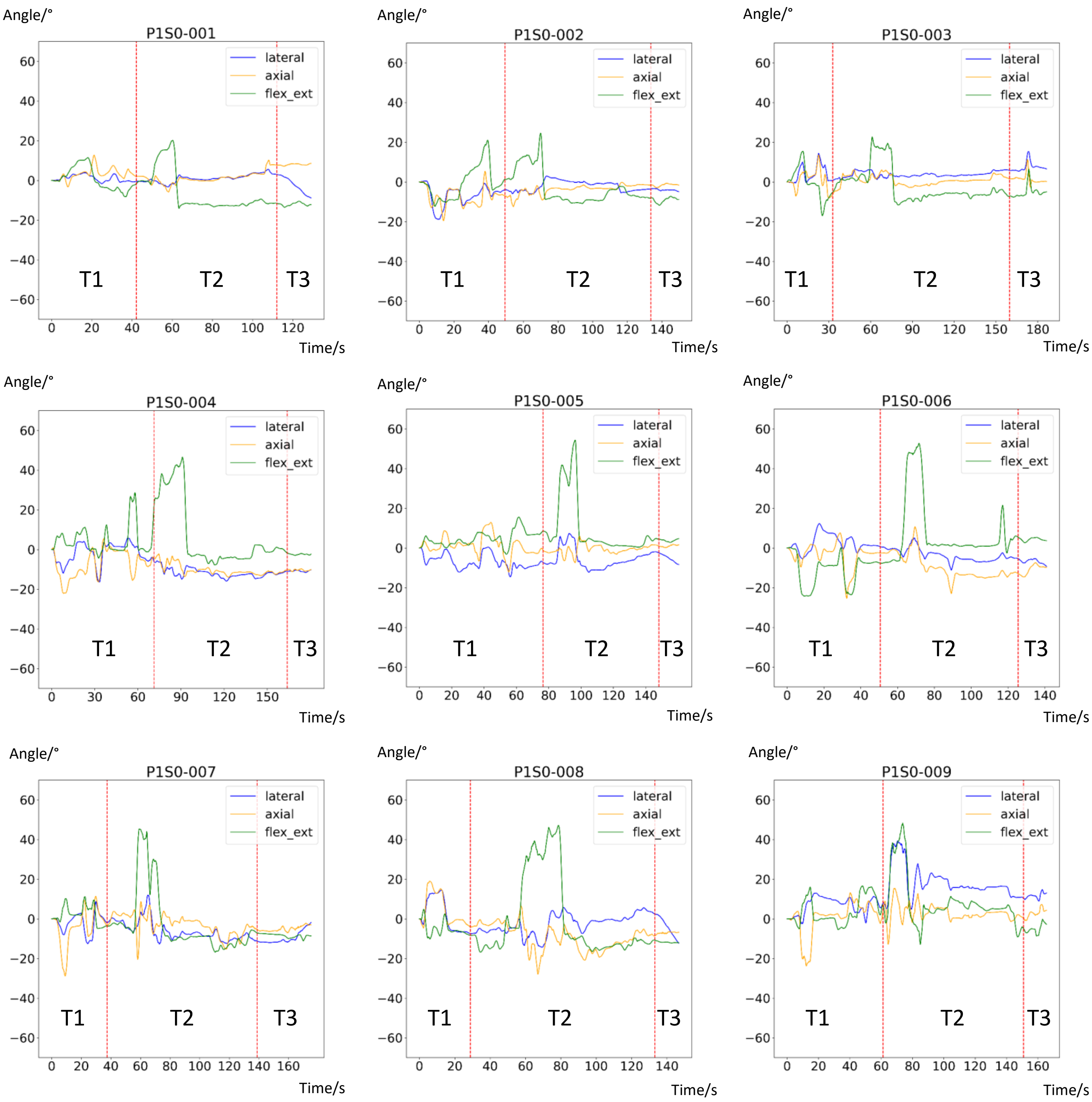

Technique P1S1: rescue sheet with rigid cervical collar

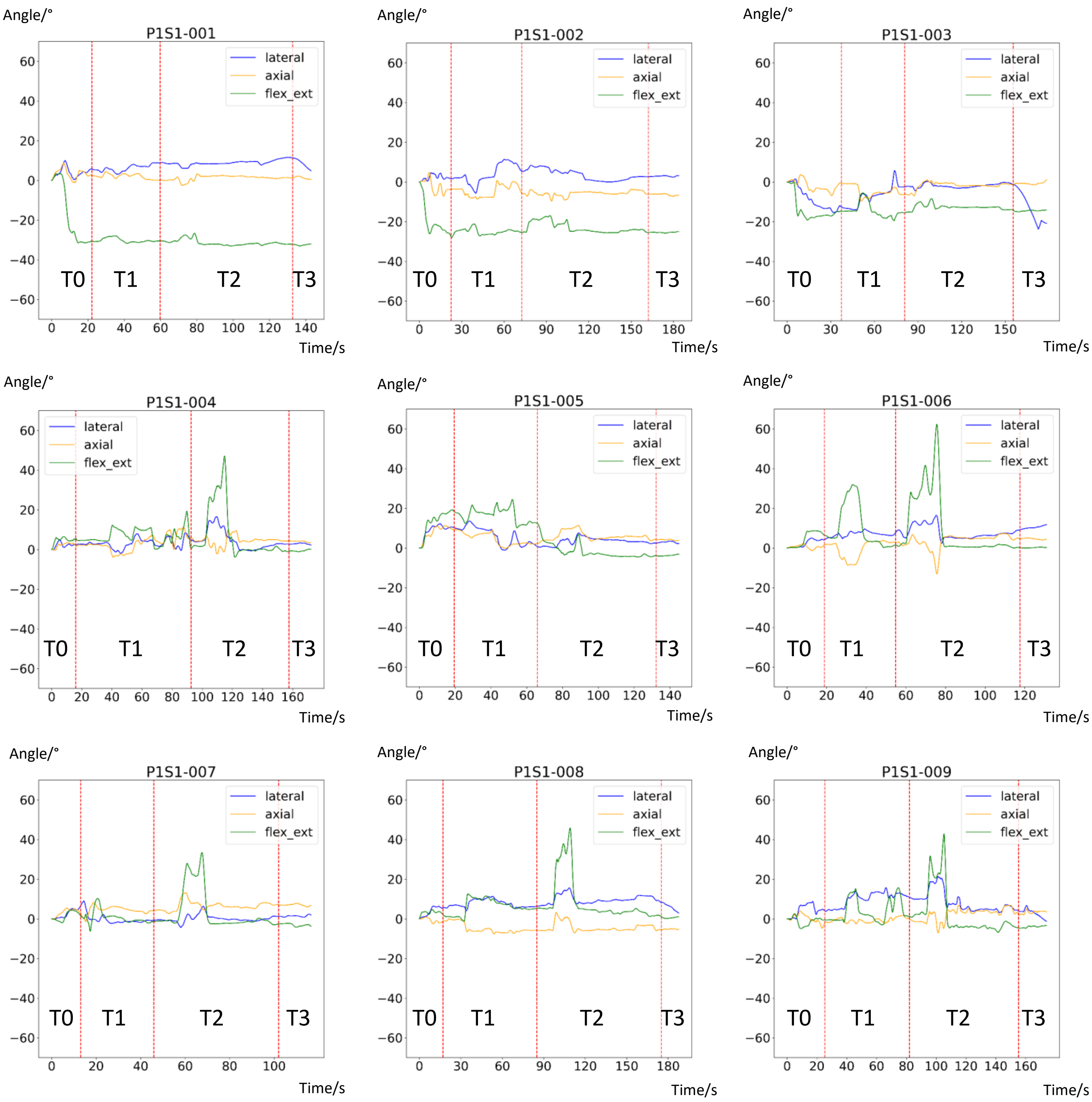

Technique P2S0: vacuum mattress without rigid cervical collar

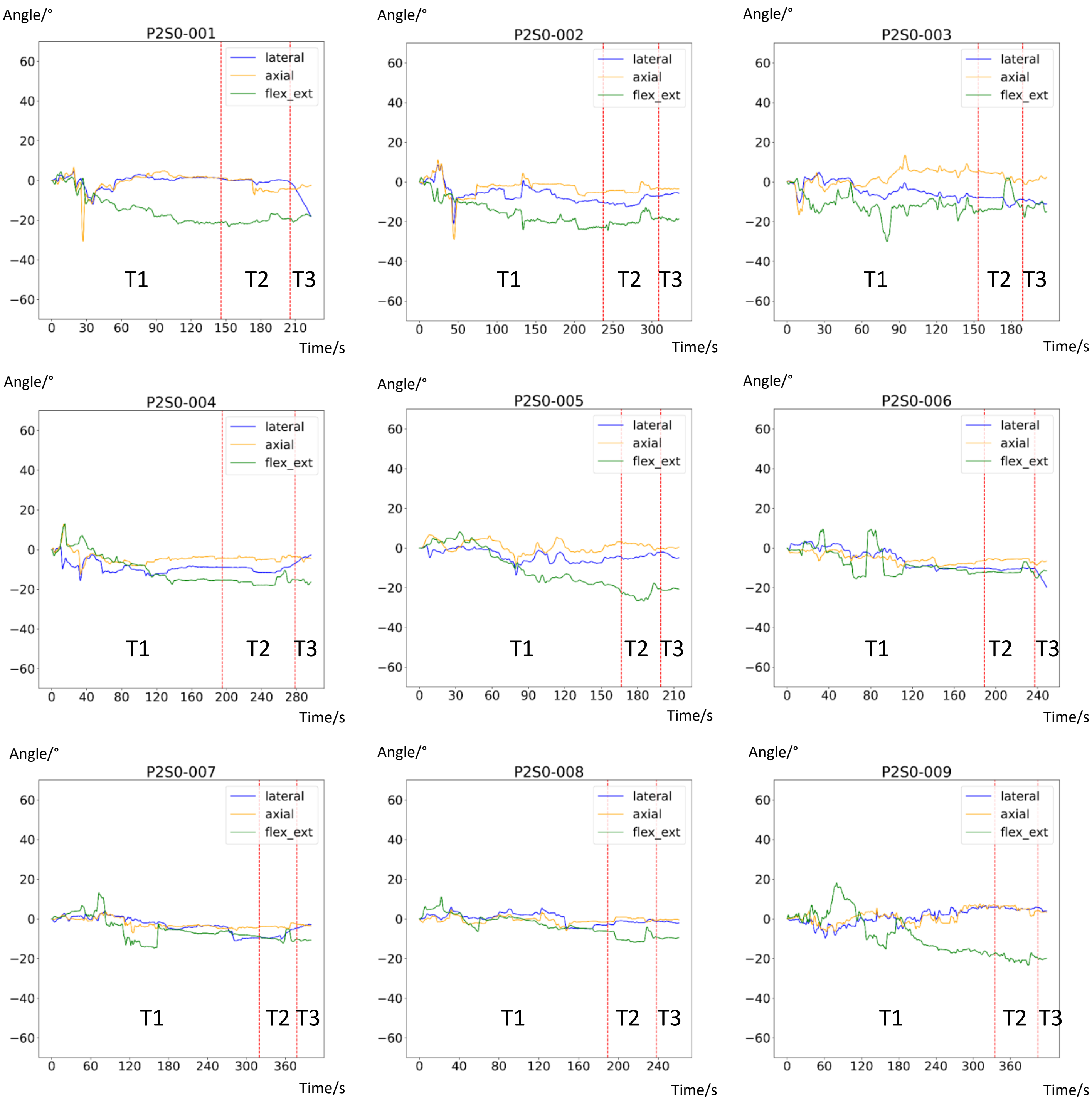

Technique P2S1: vacuum mattress with rigid cervical collar

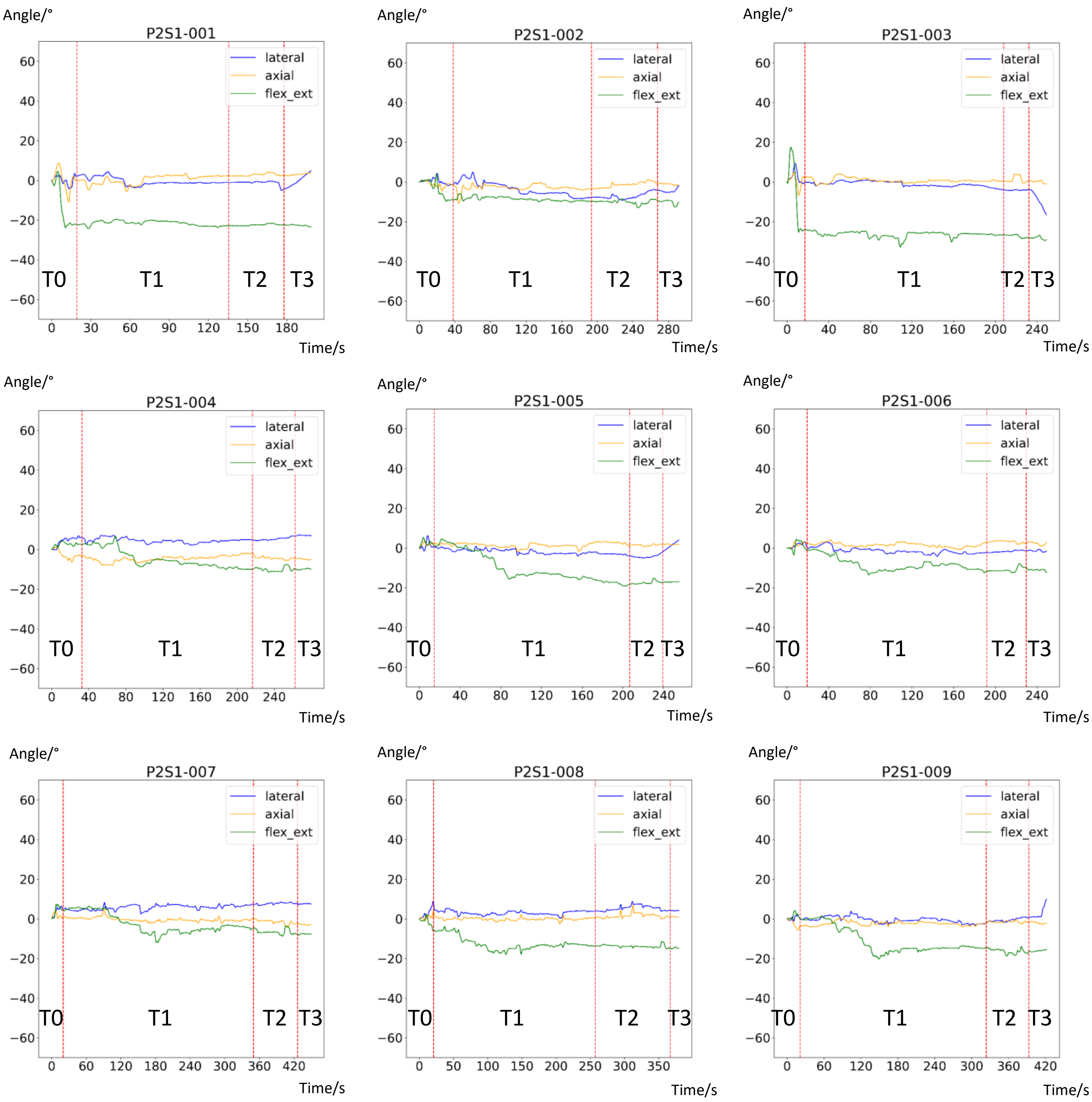

Technique P3S0: long spinal board without rigid cervical collar

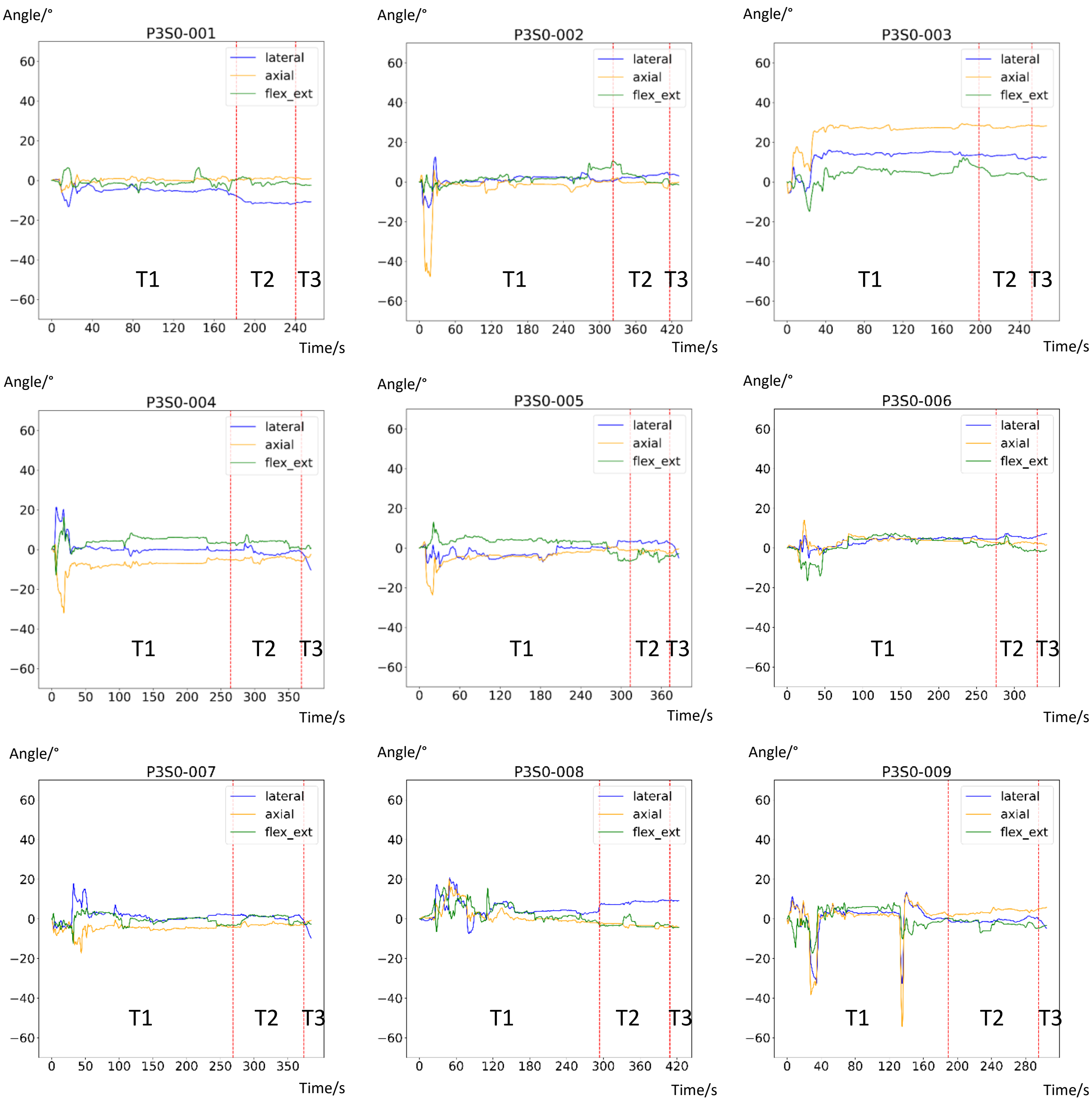

Technique P3S1: long spinal board with rigid cervical collar

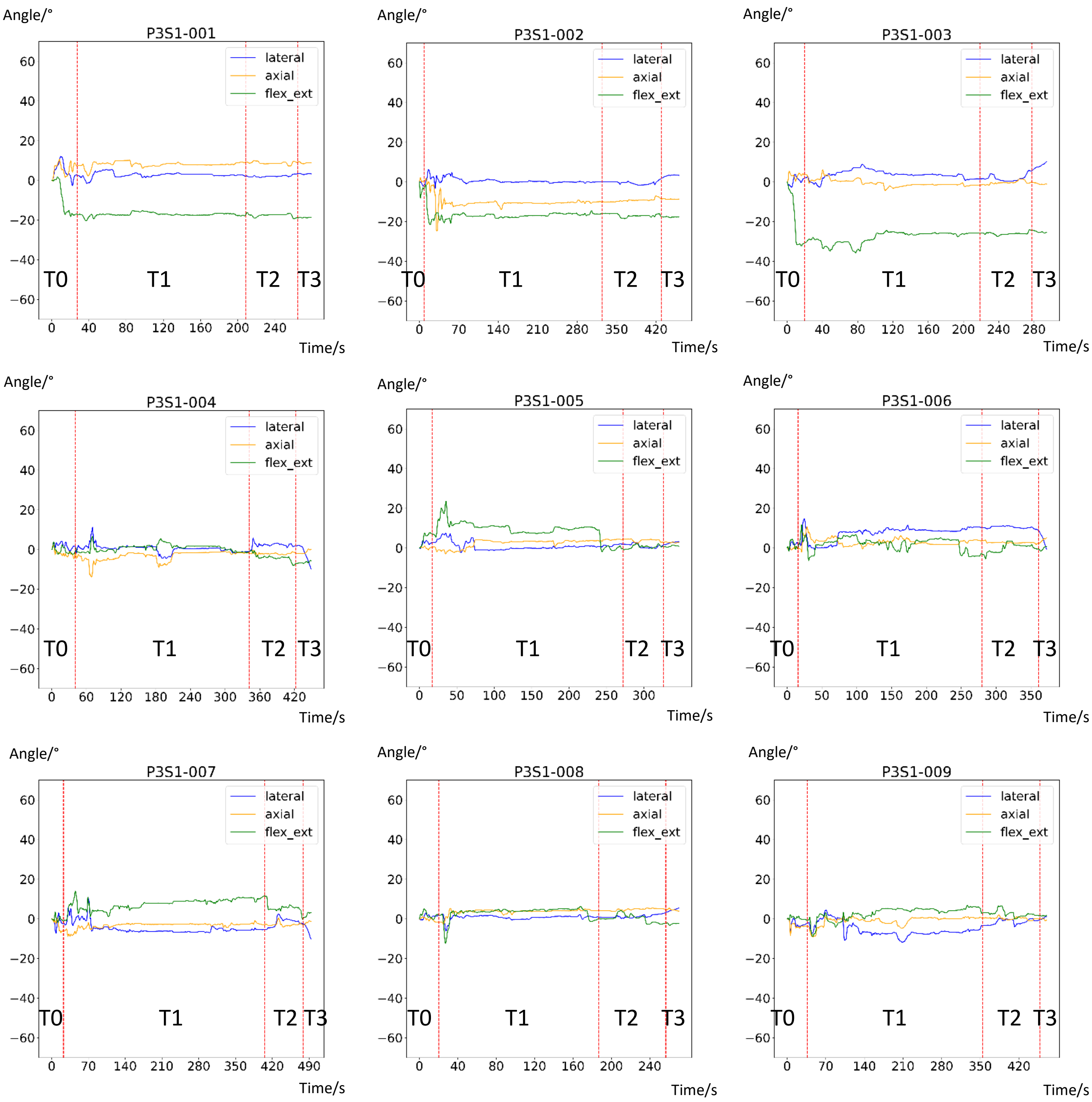

Plotted for each trial are the measured angles in degrees versus time in seconds, in yellow the angle of axial rotation about the cranio-caudal axis, in blue the lateral bending angle in the frontal plane, and in green the angle of extension/flexion in the sagittal plane.

The time is divided by the red vertical lines into the time periods T0, T1, T2 and T3, where in the first time period T0 the application of the rigid cervical collar occurs and is present only in the trials with this tool.

For each of the 6 techniques, 9 trials were performed, the first 3 each with subject 1, the others with subject 2. Although both subjects were male and similar in height and age, differences can be seen, especially in the trials without rigid cervical collar attributable to the subjects.
