# Supplementary material for: Comparison of different techniques for prehospital cervical spine immobilization: Biomechanical measurements with a wireless motion capture system
Source: PLoS One. 2023 Nov 28;18(11):e0292300. doi: 10.1371/journal.pone.0292300 (PMC10683997; doi:10.1371/journal.pone.0292300)
Supplement: S1 Table — (DOCX) [file pone.0292300.s002.docx]

**S1 Table**

**Duration analysis**

|  | **P1S0** | **P1S1** | **P2S0** | **P2S1** | **P3S0** | **P2S1** |
| --- | --- | --- | --- | --- | --- | --- |
| **P1S0** |  | 1.000 | <0.001 |  | <0.001 |  |
| **P1S1** | 1.000 |  |  | <0.001 |  | <0.001 |
| **P2S0** | <0.001 |  |  | 0.605 | 0.077 |  |
| **P2S1** |  | <0.001 | 0.605 |  |  | 0.077 |
| **P3S0** | <0.001 |  | 0.077 |  |  | 0.436 |
| **P2S1** |  | <0.001 |  | 0.077 | 0.436 |  |

Here, the total durations Ttotal of the experimental configurations were compared pairwise by using the Mann-Whitney U test, in accordance with Table 3 of the manuscript. The values in the table are the determined p-values. Values below the significance level of 0.05 chosen in the study are marked in red.
