# Supplementary material for: Comparison of different techniques for prehospital cervical spine immobilization: Biomechanical measurements with a wireless motion capture system
Source: PLoS One. 2023 Nov 28;18(11):e0292300. doi: 10.1371/journal.pone.0292300 (PMC10683997; doi:10.1371/journal.pone.0292300)
Supplement: S2 Table — (DOCX) [file pone.0292300.s003.docx]

**S2 Table**

**Analysis of axial rotation around the cranio-caudal axis**

**Time interval T1:**

| **mean maximum absolute angle** | **P1S0** | **P1S1** | **P2S0** | **P2S1** | **P3S0** | **P3S1** |
| --- | --- | --- | --- | --- | --- | --- |
| **P1S0** |  | <0.0001 | 0.0583 |  | 0.0117 |  |
| **P1S1** | <0.0001 |  |  | 0.4079 |  | 0.3751 |
| **P2S0** | 0.0583 |  |  | 0.0030 | <0.0001 |  |
| **P2S1** |  | 0.4079 | 0.0030 |  |  | 0.0882 |
| **P3S0** | 0.0117 |  | <0.0001 |  |  | <0.0001 |
| **P3S1** |  | 0.3751 |  | 0.0882 | <0.0001 |  |

| **mean angle range** | **P1S0** | **P1S1** | **P2S0** | **P2S1** | **P3S0** | **P3S1** |
| --- | --- | --- | --- | --- | --- | --- |
| **P1S0** |  | <0.0001 | 0.0433 |  | 0.1471 |  |
| **P1S1** | <0.0001 |  |  | 0.4292 |  | 0.3928 |
| **P2S0** | 0.0433 |  |  | <0.0001 | 0.0007 |  |
| **P2S1** |  | 0.4292 | <0.0001 |  |  | 0.1017 |
| **P3S0** | 0.1471 |  | 0.0007 |  |  | <0.0001 |
| **P3S1** |  | 0.3928 |  | 0.1017 | <0.0001 |  |

**Time interval T2:**

| **mean maximum absolute angle** | **P1S0** | **P1S1** | **P2S0** | **P2S1** | **P3S0** | **P3S1** |
| --- | --- | --- | --- | --- | --- | --- |
| **P1S0** |  | 0.0362 | 0.0011 |  | 0.0057 |  |
| **P1S1** | 0.0362 |  |  | 0.0855 |  | 0.1710 |
| **P2S0** | 0.0011 |  |  | 0.6198 | 0.5956 |  |
| **P2S1** |  | 0.0855 | 0.6198 |  |  | 0.7212 |
| **P3S0** | 0.0057 |  | 0.5956 |  |  | 0.5022 |
| **P3S1** |  | 0.1710 |  | 0.7212 | 0.5022 |  |

| **mean angle range** | **P1S0** | **P1S1** | **P2S0** | **P2S1** | **P3S0** | **P3S1** |
| --- | --- | --- | --- | --- | --- | --- |
| **P1S0** |  | 0.0007 | <0.0001 |  | <0.0001 |  |
| **P1S1** | 0.0007 |  |  | 0.0189 |  | 0.0116 |
| **P2S0** | <0.0001 |  |  | 0.6984 | 0.6308 |  |
| **P2S1** |  | 0.0189 | 0.6984 |  |  | 0.8521 |
| **P3S0** | <0.0001 |  | 0.6308 |  |  | 0.9257 |
| **P3S1** |  | 0.0116 |  | 0.8521 | 0.9257 |  |

**Time interval T3:**

| **mean maximum absolute angle** | **P1S0** | **P1S1** | **P2S0** | **P2S1** | **P3S0** | **P3S1** |
| --- | --- | --- | --- | --- | --- | --- |
| **P1S0** |  | 0.2544 | 0.1506 |  | 0.4841 |  |
| **P1S1** | 0.2544 |  |  | 0.4700 |  | 0.8784 |
| **P2S0** | 0.1506 |  |  | 0.6732 | 0.4572 |  |
| **P2S1** |  | 0.4700 | 0.6732 |  |  | 0.5687 |
| **P3S0** | 0.4841 |  | 0.4572 |  |  | 0.5515 |
| **P3S1** |  | 0.8784 |  | 0.5687 | 0.5515 |  |

| **mean angle range** | **P1S0** | **P1S1** | **P2S0** | **P2S1** | **P3S0** | **P3S1** |
| --- | --- | --- | --- | --- | --- | --- |
| **P1S0** |  | 0.2926 | 0.3208 |  | 0.2710 |  |
| **P1S1** | 0.2926 |  |  | 0.9091 |  | 0.9813 |
| **P2S0** | 0.3208 |  |  | 0.8616 | 0.9132 |  |
| **P2S1** |  | 0.9190 | 0.8616 |  |  | 0.9277 |
| **P3S0** | 0.2710 |  | 0.9132 |  |  | 0.9798 |
| **P3S1** |  | 0.9813 |  | 0.9277 | 0.9798 |  |

Both the mean absolute angles and the mean absolute maximum angles of the experimental setups were compared by using ANOVA, in accordance with Table 4 of the manuscript. The values in the table are the determined p-values. Values below the significance level of 0.05 chosen in the study are marked in red. The time intervals T1, T2 and T3 were considered individually.
