# Supplementary material for: Comparison of different techniques for prehospital cervical spine immobilization: Biomechanical measurements with a wireless motion capture system
Source: PLoS One. 2023 Nov 28;18(11):e0292300. doi: 10.1371/journal.pone.0292300 (PMC10683997; doi:10.1371/journal.pone.0292300)
Supplement: S3 Table — (DOCX) [file pone.0292300.s004.docx]

**S3 Table**

**Analysis of lateral bending in the frontal plane**

**Time interval T1:**

| **mean maximum absolute angle** | **P1S0** | **P1S1** | **P2S0** | **P2S1** | **P3S0** | **P3S1** |
| --- | --- | --- | --- | --- | --- | --- |
| **P1S0** |  | 0.3916 | 0.6768 |  | 0.1496 |  |
| **P1S1** | 0.3916 |  |  | 0.0191 |  | 0.3581 |
| **P2S0** | 0.6768 |  |  | 0.0057 | 0.0644 |  |
| **P2S1** |  | 0.0191 | 0.0057 |  |  | 0.1473 |
| **P3S0** | 0.1496 |  | 0.0644 |  |  | 0.0017 |
| **P3S1** |  | 0.3581 |  | 0.1473 | 0.0017 |  |

| **mean angle range** | **P1S0** | **P1S1** | **P2S0** | **P2S1** | **P3S0** | **P3S1** |
| --- | --- | --- | --- | --- | --- | --- |
| **P1S0** |  | 0.0263 | 0.9535 |  | 0.0144 |  |
| **P1S1** | 0.0263 |  |  | 0.0722 |  | 0.7332 |
| **P2S0** | 0.9535 |  |  | <0.0001 | 0.0123 |  |
| **P2S1** |  | 0.0722 | <0.0001 |  |  | 0.0333 |
| **P3S0** | 0.0144 |  | 0.0123 |  |  | <0.0001 |
| **P3S1** |  | 0.7332 |  | 0.0333 | <0.0001 |  |

**Time interval T2:**

| **mean maximum absolute angle** | **P1S0** | **P1S1** | **P2S0** | **P2S1** | **P3S0** | **P3S1** |
| --- | --- | --- | --- | --- | --- | --- |
| **P1S0** |  | 0.4525 | 0.0250 |  | 0.0026 |  |
| **P1S1** | 0.4525 |  |  | 0.0091 |  | 0.0027 |
| **P2S0** | 0.0250 |  |  | 0.2565 | 0.4150 |  |
| **P2S1** |  | 0.0091 | 0.2565 |  |  | 0.6768 |
| **P3S0** | 0.0026 |  | 0.4150 |  |  | 0.4605 |
| **P3S1** |  | 0.0027 |  | 0.6768 | 0.4605 |  |

| **mean angle range** | **P1S0** | **P1S1** | **P2S0** | **P2S1** | **P3S0** | **P3S1** |
| --- | --- | --- | --- | --- | --- | --- |
| **P1S0** |  | 0.0169 | <0.0001 |  | <0.0001 |  |
| **P1S1** | 0.0169 |  |  | 0.0024 |  | 0.0105 |
| **P2S0** | <0.0001 |  |  | 0.7311 | 0.8711 |  |
| **P2S1** |  | 0.0024 | 0.7311 |  |  | 0.6163 |
| **P3S0** | <0.0001 |  | 0.8711 |  |  | 0.7490 |
| **P3S1** |  | 0.0105 |  | 0.6163 | 0.7490 |  |

**Time interval T3:**

| **mean maximum absolute angle** | **P1S0** | **P1S1** | **P2S0** | **P2S1** | **P3S0** | **P3S1** |
| --- | --- | --- | --- | --- | --- | --- |
| **P1S0** |  | 0.2767 | 0.4468 |  | 0.2832 |  |
| **P1S1** | 0.2767 |  |  | 0.5636 |  | 0.3833 |
| **P2S0** | 0.4468 |  |  | 0.3653 | 0.7532 |  |
| **P2S1** |  | 0.5636 | 0.3653 |  |  | 0.7677 |
| **P3S0** | 0.2832 |  | 0.7532 |  |  | 0.3754 |
| **P3S1** |  | 0.3833 |  | 0.7677 | 0.3754 |  |

| **mean angle range** | **P1S0** | **P1S1** | **P2S0** | **P2S1** | **P3S0** | **P3S1** |
| --- | --- | --- | --- | --- | --- | --- |
| **P1S0** |  | 0.4804 | 0.3240 |  | 0.1574 |  |
| **P1S1** | 0.4804 |  |  | 0.7691 |  | 0.7022 |
| **P2S0** | 0.3240 |  |  | 0.9909 | 0.6655 |  |
| **P2S1** |  | 0.7691 | 0.9909 |  |  | 0.9292 |
| **P3S0** | 0.1574 |  | 0.6655 |  |  | 0.7397 |
| **P3S1** |  | 0.7022 |  | 0.9292 | 0.7397 |  |

Both the mean absolute angles and the mean absolute maximum angles of the experimental setups were compared by using ANOVA, according to Table 5 of the manuscript. The values in the table are the determined p-values. Values below the significance level of 0.05 chosen in the study are marked in red. The time intervals T1, T2 and T3 were considered individually.
