# Supplementary material for: Comparison of different techniques for prehospital cervical spine immobilization: Biomechanical measurements with a wireless motion capture system
Source: PLoS One. 2023 Nov 28;18(11):e0292300. doi: 10.1371/journal.pone.0292300 (PMC10683997; doi:10.1371/journal.pone.0292300)
Supplement: S4 Table — (DOCX) [file pone.0292300.s005.docx]

**S4 Table**

**Analysis of flexion and extension in the sagittal plane**

**Time interval T1:**

| **mean minimum angle** | **P1S0** | **P1S1** | **P2S0** | **P2S1** | **P3S0** | **P3S1** |
| --- | --- | --- | --- | --- | --- | --- |
| **P1S0** |  | 0.6534 | 0.0292 |  | 0.9391 |  |
| **P1S1** | 0.6534 |  |  | 0.0248 |  | 0.4112 |
| **P2S0** | 0.0292 |  |  | 0.7014 | 0.0351 |  |
| **P2S1** |  | 0.0248 | 0.7014 |  |  | 0.1491 |
| **P3S0** | 0.9391 |  | 0.0351 |  |  | 0.7661 |
| **P3S1** |  | 0.4112 |  | 0.1491 | 0.7661 |  |

| **mean maximum angle** | **P1S0** | **P1S1** | **P2S0** | **P2S1** | **P3S0** | **P3S1** |
| --- | --- | --- | --- | --- | --- | --- |
| **P1S0** |  | 0.1224 | 0.2567 |  | 0.3827 |  |
| **P1S1** | 0.1224 |  |  | 0.0777 |  | 0.5529 |
| **P2S0** | 0.2567 |  |  | 0.0301 | 0.7924 |  |
| **P2S1** |  | 0.0777 | 0.0301 |  |  | 0.2377 |
| **P3S0** | 0.3827 |  | 0.7924 |  |  | 0.2046 |
| **P3S1** |  | 0.5529 |  | 0.2377 | 0.2046 |  |

| **mean angle range** | **P1S0** | **P1S1** | **P2S0** | **P2S1** | **P3S0** | **P3S1** |
| --- | --- | --- | --- | --- | --- | --- |
| **P1S0** |  | 0.0023 | 0.5803 |  | 0.1679 |  |
| **P1S1** | 0.0023 |  |  | 0.6519 |  | 0.9370 |
| **P2S0** | 0.5803 |  |  | <0.0001 | 0.0548 |  |
| **P2S1** |  | 0.6519 | <0.0001 |  |  | 0.7098 |
| **P3S0** | 0.1679 |  | 0.0548 |  |  | 0.0724 |
| **P3S1** |  | 0.9370 |  | 0.7098 | 0.0724 |  |

**Time interval T2:**

| **mean minimum angle** | **P1S0** | **P1S1** | **P2S0** | **P2S1** | **P3S0** | **P3S1** |
| --- | --- | --- | --- | --- | --- | --- |
| **P1S0** |  | 0.8803 | 0.0385 |  | 0.0423 |  |
| **P1S1** | 0.8803 |  |  | 0.1051 |  | 0.7923 |
| **P2S0** | 0.0385 |  |  | 0.5410 | <0.0001 |  |
| **P2S1** |  | 0.1051 | 0.5410 |  |  | 0.0604 |
| **P3S0** | 0.0423 |  | <0.0001 |  |  | 0.1037 |
| **P3S1** |  | 0.7923 |  | 0.0604 | 0.1037 |  |

| **mean maximum angle** | **P1S0** | **P1S1** | **P2S0** | **P2S1** | **P3S0** | **P3S1** |
| --- | --- | --- | --- | --- | --- | --- |
| **P1S0** |  | 0.0014 | <0.0001 |  | <0.0001 |  |
| **P1S1** | 0.0014 |  |  | <0.0001 |  | <0.0001 |
| **P2S0** | <0.0001 |  |  | 0.6123 | 0.0193 |  |
| **P2S1** |  | <0.0001 | 0.6123 |  |  | 0.1206 |
| **P3S0** | <0.0001 |  | 0.0193 |  |  | 0.1892 |
| **P3S1** |  | <0.0001 |  | 0.1206 | 0.1892 |  |

| **mean angle range** | **P1S0** | **P1S1** | **P2S0** | **P2S1** | **P3S0** | **P3S1** |
| --- | --- | --- | --- | --- | --- | --- |
| **P1S0** |  | <0.0001 | <0.0001 |  | <0.0001 |  |
| **P1S1** | <0.0001 |  |  | <0.0001 |  | <0.0001 |
| **P2S0** | <0.0001 |  |  | 0.1267 | 0.5240 |  |
| **P2S1** |  | <0.0001 | 0.1267 |  |  | 0.6097 |
| **P3S0** | <0.0001 |  | 0.5240 |  |  | 0.6982 |
| **P3S1** |  | <0.0001 |  | 0.6097 | 0.6982 |  |

**Time interval T3:**

| **mean minimum angle** | **P1S0** | **P1S1** | **P2S0** | **P2S1** | **P3S0** | **P3S1** |
| --- | --- | --- | --- | --- | --- | --- |
| **P1S0** |  | 0.4261 | 0.0082 |  | 0.2541 |  |
| **P1S1** | 0.4261 |  |  | 0.1185 |  | 0.5917 |
| **P2S0** | 0.0082 |  |  | 0.7450 | 0.0002 |  |
| **P2S1** |  | 0.1185 | 0.7450 |  |  | 0.0371 |
| **P3S0** | 0.2541 |  | 0.0002 |  |  | 0.1623 |
| **P3S1** |  | 0.5917 |  | 0.0371 | 0.1623 |  |

| **mean maximum angle** | **P1S0** | **P1S1** | **P2S0** | **P2S1** | **P3S0** | **P3S1** |
| --- | --- | --- | --- | --- | --- | --- |
| **P1S0** |  | 0.2713 | 0.0386 |  | 0.7893 |  |
| **P1S1** | 0.2713 |  |  | 0.3295 |  | 0.7341 |
| **P2S0** | 0.0386 |  |  | 0.9916 | 0.0200 |  |
| **P2S1** |  | 0.3295 | 0.9916 |  |  | 0.1897 |
| **P3S0** | 0.7893 |  | 0.0200 |  |  | 0.3038 |
| **P3S1** |  | 0.7341 |  | 0.1897 | 0.3038 |  |

| **mean angle range** | **P1S0** | **P1S1** | **P2S0** | **P2S1** | **P3S0** | **P3S1** |
| --- | --- | --- | --- | --- | --- | --- |
| **P1S0** |  | 0.3352 | 0.6108 |  | 0.4079 |  |
| **P1S1** | 0.3352 |  |  | 0.9112 |  | 0.9774 |
| **P2S0** | 0.6108 |  |  | 0.7299 | 0.7491 |  |
| **P2S1** |  | 0.9112 | 0.7299 |  |  | 0.8888 |
| **P3S0** | 0.4079 |  | 0.7491 |  |  | 0.8688 |
| **P3S1** |  | 0.9774 |  | 0.8888 | 0.8688 |  |

Both the mean absolute angles and the mean minimum and maximum angles of the experimental setups were compared by using ANOVA, according to Table 6 of the manuscript. The values in the table are the determined p-values. Values below the significance level of 0.05 chosen in the study are marked in red. The time intervals T1, T2 and T3 were considered individually.
