# Supplementary material for: Comparison of different techniques for prehospital cervical spine immobilization: Biomechanical measurements with a wireless motion capture system
Source: PLoS One. 2023 Nov 28;18(11):e0292300. doi: 10.1371/journal.pone.0292300 (PMC10683997; doi:10.1371/journal.pone.0292300)
Supplement: S1 File — (DOCX) [file pone.0292300.s013.docx]

{

"cells": [

{

"cell_type": "code",

"execution_count": 2,

"metadata": {},

"outputs": [],

"source": [

"import pandas as pd\n",

"import numpy as np\n",

"import os\n",

"import matplotlib.pyplot as plt\n",

"import scipy.ndimage\n",

"import concurrent.futures\n",

"\n",

"# set constants for the figure\n",

"Y_LIM_MAX = 70\n",

"VELOCITY_Y_LIM_MAX = 40\n",

"ACC_Y_LIM_MAX = 125\n",

"\n",

"# set label font size\n",

"plt.rc('font', size=24)\n",

"\n",

"# set true, if x axis should be in seconds instead of frames\n",

"X_SCALE_IN_SECONDS = True\n",

"\n",

"# X hz to smooth the data over\n",

"SMOOTH_HZ = .5"

]

},

{

"cell_type": "code",

"execution_count": 3,

"metadata": {},

"outputs": [],

"source": [

"def get_versuch_from_filepath(file_path):\n",

" return file_path.replace(\"data/\", \"\").replace(\".csv\", \"\")\n",

"\n",

"\n",

"def get_df_from_file(file_path):\n",

" # read file, rename columns\n",

" with open(file_path, 'r') as f:\n",

" df = pd.read_csv(f, sep=';', decimal=\",\", index_col=0)\n",

" df.rename(columns={\n",

" df.columns[0]: 'lateral',\n",

" df.columns[1]: 'axial',\n",

" df.columns[2]: 'flex_ext'\n",

" }, inplace=True)\n",

" # flips lateral values\n",

" df['lateral'] = df['lateral'] * -1\n",

" return df\n",

"\n",

"\n",

"def smooth_data(df):\n",

" df = df.copy()\n",

" lateral_array = df['lateral'].to_numpy()\n",

" axial_array = df['axial'].to_numpy()\n",

" flex_ext_array = df['flex_ext'].to_numpy()\n",

"\n",

" filter_size = int(round(240 / SMOOTH_HZ)) # edit quotient to smooth array over X frames, where X is the desired framerate (default: 5, meaning 5hz)\n",

"\n",

" # smooth the arrays with a 1-D uniform filter\n",

" lateral_array_smoothed = scipy.ndimage.uniform_filter1d(\n",

" lateral_array, filter_size, mode='nearest')\n",

" axial_array_smoothed = scipy.ndimage.uniform_filter1d(\n",

" axial_array, filter_size, mode='nearest')\n",

" flex_ext_array_smoothed = scipy.ndimage.uniform_filter1d(\n",

" flex_ext_array, filter_size, mode='nearest')\n",

"\n",

" # add smoothed arrays to dataframe\n",

" df['lateral_smoothed'] = lateral_array_smoothed\n",

" df['axial_smoothed'] = axial_array_smoothed\n",

" df['flex_ext_smoothed'] = flex_ext_array_smoothed\n",

"\n",

" return df\n",

"\n",

"\n",

"def transform_data(df, versuch, timestamps):\n",

" df = df.copy()\n",

" v = timestamps[timestamps['Versuch'] == versuch]\n",

" nn = int(v['NN_frame'])\n",

" nn_frame = df.iloc[nn]\n",

"\n",

" # center data around NN frame by subtracting NN frame from respective data\n",

" df['lateral_smoothed_averaged'] = df['lateral_smoothed'] - \\\n",

" nn_frame['lateral_smoothed']\n",

" df['axial_smoothed_averaged'] = df['axial_smoothed'] - \\\n",

" nn_frame['axial_smoothed']\n",

" df['flex_ext_smoothed_averaged'] = df['flex_ext_smoothed'] - \\\n",

" nn_frame['flex_ext_smoothed']\n",

" return df\n",

"\n",

"\n",

"def smooth_transform_data(df, versuch, timestamps):\n",

" df = df.copy()\n",

" df = smooth_data(df)\n",

" df = transform_data(df, versuch, timestamps)\n",

" return df\n",

"\n",

"\n",

"def plot_df(df, versuch, timestamps):\n",

" z = df['lateral_smoothed_averaged'].to_numpy()\n",

" x = df['axial_smoothed_averaged'].to_numpy()\n",

" y = df['flex_ext_smoothed_averaged'].to_numpy()\n",

" v = timestamps[timestamps['Versuch'] == versuch]\n",

"\n",

" # save prepared data to csv\n",

" pd.DataFrame({'lateral': z, 'axial': x, 'flex_ext': y}).to_csv(\n",

" f'output/parsed/{versuch}.csv', sep=';', decimal=\",\")\n",

"\n",

" # set up figure\n",

" fig, ax = plt.subplots(figsize=(10, 10), dpi=300)\n",

" ax.set_ylim(Y_LIM_MAX * -1, Y_LIM_MAX)\n",

" x_axis = np.arange(len(z))\n",

" # read timestamps\n",

" m0 = v['M0']\n",

" m1 = v['M1']\n",

" m2 = v['M2']\n",

" m3 = v['M3']\n",

" m4 = v['M4']\n",

"\n",

" # trim data to not include data starting from m3\n",

" z = z[:int(m3)]\n",

" x = x[:int(m3)]\n",

" y = y[:int(m3)]\n",

" x_axis = x_axis[:int(m3)]\n",

"\n",

" # convert frames to seconds if set\n",

" if X_SCALE_IN_SECONDS:\n",

" x_axis_s = np.around(len(x) / 240, 0)\n",

" labels = np.around(np.linspace(0, x_axis_s, num=8), -1)\n",

" labels = np.arange(0, x_axis_s, step=labels[1])\n",

" locs = labels.copy() * 240\n",

" labels = [str(int(x)) for x in labels]\n",

" ax.set_xticks(locs)\n",

" ax.set_xticklabels(labels)\n",

"\n",

"\n",

" # plot data\n",

" z_line = ax.plot(x_axis, z, label='lateral', color=\"blue\")\n",

" x_line = ax.plot(x_axis, x, label='axial', color=\"orange\")\n",

" y_line = ax.plot(x_axis, y, label='flex_ext', color= \"green\")\n",

" legend = ax.legend()\n",

" ymin, ymax = ax.get_ylim()\n",

" # plot markers divinding timeframes\n",

" if not m0.hasnans:\n",

" ax.vlines(m0, ymin=ymin, ymax=ymax,\n",

" color='r', label='M0', linestyle='--')\n",

" ax.vlines(m1, ymin=ymin, ymax=ymax,\n",

" color='r', label='M1', linestyle='--')\n",

" ax.vlines(m2, ymin=ymin, ymax=ymax,\n",

" color='r', label='M2', linestyle='--')\n",

" # ax.vlines(m3, ymin=ymin, ymax=ymax,\n",

" # color='r', label='M3', linestyle='--')\n",

" # ax.vlines(m4, ymin=ymin, ymax=ymax,\n",

" # color='r', label='M4', linestyle='--')\n",

"\n",

" ax.set_title(versuch)\n",

" fig.savefig(f\"output/plots/{versuch}.png\", facecolor='w', edgecolor='w')\n",

"\n",

" legend.remove()\n",

" def remove_line(line):\n",

" for handle in line:\n",

" handle.remove()\n",

"\n",

" remove_line(z_line)\n",

" remove_line(x_line)\n",

" remove_line(y_line)\n",

"\n",

" z_line = ax.plot(x_axis, z, label='lateral', color=\"blue\")\n",

" fig.savefig(f\"output/plots/split/{versuch}_lateral.png\", facecolor='w', edgecolor='w')\n",

" remove_line(z_line)\n",

"\n",

" x_line = ax.plot(x_axis, x, label='axial', color=\"orange\")\n",

" fig.savefig(f\"output/plots/split/{versuch}_axial.png\", facecolor='w', edgecolor='w')\n",

" remove_line(x_line)\n",

"\n",

" y_line = ax.plot(x_axis, y, label='flex_ext', color= \"green\")\n",

" fig.savefig(f\"output/plots/split/{versuch}_flex_ext.png\", facecolor='w', edgecolor='w')\n",

" remove_line(y_line) \n",

"\n",

" plt.close()\n",

"\n",

"\n",

"def summary_data_by_timeframe(df, versuch, timestamps, save_or_return='save'):\n",

" # generate summary data for each timeframe\n",

" # read timestamps\n",

" v = timestamps[timestamps['Versuch'] == versuch]\n",

" m0 = v['M0'].to_numpy()[0]\n",

" if not np.isnan(m0):\n",

" m0 = int(m0)\n",

" m1 = int(v['M1'].to_numpy()[0])\n",

" m2 = int(v['M2'].to_numpy()[0])\n",

" m3 = int(v['M3'].to_numpy()[0])\n",

" m4 = int(v['M4'].to_numpy()[0])\n",

"\n",

" # divide data into timeframes\n",

" if not np.isnan(m0):\n",

" t0 = df.iloc[slice(0, m0)]\n",

" t1 = df.iloc[slice(m0, m1)]\n",

" else:\n",

" t1 = df.iloc[slice(0, m1)]\n",

" t2 = df.iloc[slice(m1, m2)]\n",

" t3 = df.iloc[slice(m2, m3)]\n",

" t4 = df.iloc[slice(m3, m4)]\n",

"\n",

" if not np.isnan(m0):\n",

" t0_summary = t0.iloc[:, 6:9].describe().assign(timeframe='t0')\n",

" t0_abs_summary = t0.iloc[:, 6:9].abs(\n",

" ).describe().assign(timeframe='t0_abs')\n",

" t1_summary = t1.iloc[:, 6:9].describe().assign(timeframe='t1')\n",

" t1_abs_summary = t1.iloc[:, 6:9].abs(\n",

" ).describe().assign(timeframe='t1_abs')\n",

" t2_summary = t2.iloc[:, 6:9].describe().assign(timeframe='t2')\n",

" t2_abs_summary = t2.iloc[:, 6:9].abs(\n",

" ).describe().assign(timeframe='t2_abs')\n",

" t3_summary = t3.iloc[:, 6:9].describe().assign(timeframe='t3')\n",

" t3_abs_summary = t3.iloc[:, 6:9].abs(\n",

" ).describe().assign(timeframe='t3_abs')\n",

" ### without t4\n",

" # if not np.isnan(m0):\n",

" # concat_df = pd.concat([t0_summary, t0_abs_summary, t1_summary, t1_abs_summary, t2_summary, t2_abs_summary, t3_summary, t3_abs_summary]).reset_index().rename(columns={\n",

" # 'index': 'metric',\n",

" # 'lateral_smoothed_averaged': 'lateral',\n",

" # 'axial_smoothed_averaged': 'axial',\n",

" # 'flex_ext_smoothed_averaged': 'flex_ext'\n",

" # })\n",

" # else:\n",

" # concat_df = pd.concat([t1_summary, t1_abs_summary, t2_summary, t2_abs_summary, t3_summary, t3_abs_summary]).reset_index().rename(columns={\n",

" # 'index': 'metric',\n",

" # 'lateral_smoothed_averaged': 'lateral',\n",

" # 'axial_smoothed_averaged': 'axial',\n",

" # 'flex_ext_smoothed_averaged': 'flex_ext'\n",

" # })\n",

"\n",

" ### with t4\n",

" t4_summary = t4.iloc[:, 6:9].describe().assign(timeframe='t4')\n",

" t4_abs_summary = t4.iloc[:, 6:9].abs(\n",

" ).describe().assign(timeframe='t4_abs')\n",

" if not np.isnan(m0):\n",

" concat_df = pd.concat([t0_summary, t0_abs_summary, t1_summary, t1_abs_summary, t2_summary, t2_abs_summary, t3_summary, t3_abs_summary, t4_summary, t4_abs_summary]).reset_index().rename(columns={\n",

" 'index': 'metric',\n",

" 'lateral_smoothed_averaged': 'lateral',\n",

" 'axial_smoothed_averaged': 'axial',\n",

" 'flex_ext_smoothed_averaged': 'flex_ext'\n",

" })\n",

" else:\n",

" concat_df = pd.concat([t1_summary, t1_abs_summary, t2_summary, t2_abs_summary, t3_summary, t3_abs_summary, t4_summary, t4_abs_summary]).reset_index().rename(columns={\n",

" 'index': 'metric',\n",

" 'lateral_smoothed_averaged': 'lateral',\n",

" 'axial_smoothed_averaged': 'axial',\n",

" 'flex_ext_smoothed_averaged': 'flex_ext'\n",

" })\n",

" concat_df.set_index(['timeframe', 'metric'], inplace=True)\n",

" if save_or_return == 'save':\n",

" concat_df.to_csv(\n",

" f'output/described/{versuch}.csv', sep=';', decimal=\",\", index=True)\n",

" elif save_or_return == 'return':\n",

" return concat_df"

]

},

{

"cell_type": "code",

"execution_count": 4,

"metadata": {},

"outputs": [

{

"name": "stdout",

"output_type": "stream",

"text": [

"Processing data/P1S0-001.csv\n",

"Processing data/P1S0-002.csv\n",

"Processing data/P1S0-003.csv\n",

"Processing data/P1S0-004.csv\n",

"Processing data/P1S0-005.csv\n",

"Processing data/P1S0-006.csv\n",

"Processing data/P1S0-007.csv\n",

"Processing data/P1S0-008.csv\n",

"Processing data/P1S0-009.csv\n",

"\n",

"Processing data/P1S1-001.csv\n",

"Processing data/P1S1-002.csv\n",

"Processing data/P1S1-003.csv\n",

"Processing data/P1S1-004.csv\n",

"Processing data/P1S1-005.csv\n",

"Processing data/P1S1-006.csv\n",

"Processing data/P1S1-007.csv\n",

"Processing data/P1S1-008.csv\n",

"Processing data/P1S1-009.csv\n",

"\n",

"Processing data/P2S0-001.csv\n",

"Processing data/P2S0-002.csv\n",

"Processing data/P2S0-003.csv\n",

"Processing data/P2S0-004.csv\n",

"Processing data/P2S0-005.csv\n",

"Processing data/P2S0-006.csv\n",

"Processing data/P2S0-007.csv\n",

"Processing data/P2S0-008.csv\n",

"Processing data/P2S0-009.csv\n",

"\n",

"Processing data/P2S1-001.csv\n",

"Processing data/P2S1-002.csv\n",

"Processing data/P2S1-003.csv\n",

"Processing data/P2S1-004.csv\n",

"Processing data/P2S1-005.csv\n",

"Processing data/P2S1-006.csv\n",

"Processing data/P2S1-007.csv\n",

"Processing data/P2S1-008.csv\n",

"Processing data/P2S1-009.csv\n",

"\n",

"Processing data/P3S0-001.csv\n",

"Processing data/P3S0-002.csv\n",

"Processing data/P3S0-003.csv\n",

"Processing data/P3S0-004.csv\n",

"Processing data/P3S0-005.csv\n",

"Processing data/P3S0-006.csv\n",

"Processing data/P3S0-007.csv\n",

"Processing data/P3S0-008.csv\n",

"Processing data/P3S0-009.csv\n",

"\n",

"Processing data/P3S1-001.csv\n",

"Processing data/P3S1-002.csv\n",

"Processing data/P3S1-003.csv\n",

"Processing data/P3S1-004.csv\n",

"Processing data/P3S1-005.csv\n",

"Processing data/P3S1-006.csv\n",

"Processing data/P3S1-007.csv\n",

"Processing data/P3S1-008.csv\n",

"Processing data/P3S1-009.csv\n"

]

}

],

"source": [

"def pipeline(file_path, timestamps):\n",

" print(f'Processing {file_path}')\n",

" versuch = get_versuch_from_filepath(file_path)\n",

" if np.isnan(timestamps[timestamps['Versuch'] == versuch]['NN_frame']).any():\n",

" print(f\"Versuch {versuch} hat keinen NN_frame\")\n",

" return\n",

" df = get_df_from_file(file_path)\n",

"\n",

" start_frame = timestamps[timestamps['Versuch'] == versuch][\"NN_frame\"].values[0]\n",

" df = df.iloc[start_frame:]\n",

" # update timestamps\n",

" timestamps.loc[timestamps['Versuch'] == versuch, 'NN_frame'] = 0\n",

" timestamps.loc[timestamps['Versuch'] == versuch, 'M0'] = timestamps.loc[timestamps['Versuch'] == versuch, 'M0'] - start_frame\n",

" timestamps.loc[timestamps['Versuch'] == versuch, 'M1'] = timestamps.loc[timestamps['Versuch'] == versuch, 'M1'] - start_frame\n",

" timestamps.loc[timestamps['Versuch'] == versuch, 'M2'] = timestamps.loc[timestamps['Versuch'] == versuch, 'M2'] - start_frame\n",

" timestamps.loc[timestamps['Versuch'] == versuch, 'M3'] = timestamps.loc[timestamps['Versuch'] == versuch, 'M3'] - start_frame\n",

" timestamps.loc[timestamps['Versuch'] == versuch, 'M4'] = timestamps.loc[timestamps['Versuch'] == versuch, 'M4'] - start_frame\n",

"\n",

" df_smoothed_transformed = smooth_transform_data(df, versuch, timestamps)\n",

" plot_df(df_smoothed_transformed, versuch, timestamps)\n",

" summary_data_by_timeframe(df_smoothed_transformed, versuch, timestamps)\n",

"\n",

"\n",

"def main():\n",

" with open(\"timestamps.csv\", \"r\", encoding=\"utf-8\") as f:\n",

" timestamps = pd.read_csv(f, sep=\";\", decimal=\",\")\n",

" files_list_paths = [f'data/{x}' for x in os.listdir(\"data\")]\n",

" files_list_paths.sort()\n",

"\n",

" # # pick single file for debugging\n",

" # pipeline(files_list_paths[0], timestamps)\n",

"\n",

" # speed up by parallelizing\n",

" with concurrent.futures.ProcessPoolExecutor() as executor:\n",

" executor.map(pipeline, files_list_paths, [\n",

" timestamps] * len(files_list_paths))\n",

"\n",

" # # single thread/process alternative\n",

" # for fp in files_list_paths:\n",

" # print(f\"Parsing {fp}\")\n",

" # pipeline(fp, timestamps)\n",

"\n",

"\n",

"if __name__ == \"__main__\":\n",

" main()\n"

]

}

],

"metadata": {

"interpreter": {

"hash": "7f1ef57c00fbcf37db90e994cd69fdf0719480a4d1619270038eba18cbe528cc"

},

"kernelspec": {

"display_name": "Python 3.9.7 ('base')",

"language": "python",

"name": "python3"

},

"language_info": {

"codemirror_mode": {

"name": "ipython",

"version": 3

},

"file_extension": ".py",

"mimetype": "text/x-python",

"name": "python",

"nbconvert_exporter": "python",

"pygments_lexer": "ipython3",

"version": "3.9.12"

},

"orig_nbformat": 4

},

"nbformat": 4,

"nbformat_minor": 2

}
